# The effects of lycopene on hepatic ischemia/reperfusion injury in rats

**DOI:** 10.1007/s10616-014-9706-3

**Published:** 2014-03-04

**Authors:** Gokhan Bayramoglu, Aysegul Bayramoglu, Yılmaz Altuner, Mustafa Uyanoglu, Suat Colak

**Affiliations:** 1 Department of Biology, Faculty of Art and Sciences, Artvin Coruh University, 08000 Artvin, Turkey; 2 Department of Midwifery, School of Health, Karabuk University, 78050 Karabuk, Turkey; 3 Department of Biology, Faculty of Art and Sciences, Eskisehir Osmangazi University, 26040 Eskisehir, Turkey

**Keywords:** Ischemia–reperfusion, Liver, Lycopene, Oxidative stress, Rat

## Abstract

There is a very little information about the protective effect of lycopene (LYC) against hepatic ischemia–reperfusion injury. The present study was designed to examine the possible protective effect of the strong antioxidant and anti-inflammatory agent, LYC, on hepatic ischemia/reperfusion injury. For this purpose, rats were subjected to 45 min of hepatic ischemia followed by 60 min of reperfusion period. LYC at the doses of 2.5 and 5 mg/kg body weight (bw) were injected intraperitoneally, 60 min prior to ischemia. Upon sacrification, hepatic tissue samples were used for the measurement of catalase (CAT) activity and malondialdehyde (MDA) levels. Also, aspartate aminotransferase (AST), alanine aminotransferase (ALT) and lactate dehydrogenase (LDH) were assayed in serum samples. As a result of the use of LYC at the doses of 2.5 and 5 mg/kg bw; while improvements of the ALT, AST, LDH and MDA values were partial and dose-dependent, the improvement of CAT activity was total and dose-independent (*p* < 0.05). Our findings suggest that LYC has a protective effect against ischemia/reperfusion injury on the liver.

## Introduction

Liver ischemia–reperfusion (I/R) is a serious pathologic process encountered in a number of clinical syndromes including liver transplantation, liver resection, trauma and hemorrhagic shock (Okatani et al. 2003; Jia et al. 2008; Bi et al. 2008). Ischemia and perfusion leads to oxidative stress that is characterized by an imbalance between reactive oxygen species (ROS) and the anti-oxidative defense system. The reperfusion of ischemic tissue has been shown to worsen acute ischemic injuries by releasing ROS (Akman et al. 2010).

Accumulation of ROS may easily be dealt with by endogenous anti-oxidative systems, such as catalase (CAT), superoxide dismutase (SOD) (Wang et al. 2011; Polat et al. 2008), glutathione peroxidase (GPx) and glutathione (GSH), because they already exist at low concentrations in the liver. Generated ROS, in turn, provoke damage to lipids and proteins eventually leading to liver damage. For these reasons, treatment with exogenous antioxidants, particularly in the early stages of reperfusion, significantly reduces liver I/R injury (Wang et al. 2011).

**Fig. 1:**
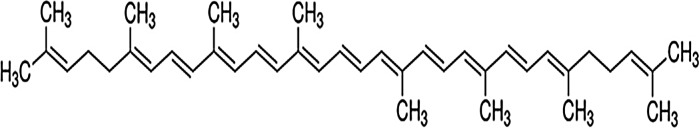
Chemical structure of lycopene

Carotenoids prevent oxidative injury in biological systems such as damage to DNA molecules, proteins, cell membrane, lipids and other structures in the cell. Lycopene (LYC) is a “nonprovitamin A carotenoid” (Aydin and Çelik 2012). The chemical structure of LYC (Fig. 1) has a 40-carbon acyclic carotenoid with 11 linearly arranged conjugated double bonds (Tang et al. 2007). LYC is one dietary carotenoid found in fruits such as papaya, fresh ripe tomato, guava, grapefruit and watermelon (Yaping et al. 2002). This carotenoid is a potent anticancer, anti-inflammatory, antiproliferative, neuroprotective, cognition enhancer and hypocholesterolemic agent (Kuhad et al. 2008). In addition, it has been ranked as being most potent among the following antioxidants: lycopene > α-tocopherol > α-carotene > β-cryptoxanthin > zeaxanthin = β-carotene > lutein. Moreover, several studies suggest that LYC is a more potent scavenger of oxygen radicals than other major dietary carotenoids (Alshatwi et al. 2010). LYC, because of its high number of conjugated double bonds, has been declared to exhibit higher singlet oxygen quenching ability compared to β-carotene or α-tocopherol and to act as a potent antioxidant, preventing the oxidative damage of critical biomolecules including lipids, proteins and DNA (Rios et al. 2009; Palozza et al. 2010). Also, LYC has been under marked investigation for its anti-oxidant benefits in treating various chronic human diseases like cancer, cardiovascular diseases, osteoporosis and diabetes (Kuhad et al. 2008).

To our knowledge, there is a very little information about this carotenoid against hepatic I/R injury in rats. For this aim, We aimed to examine the role of LYC at the doses of 2.5 and 5 mg/kg bw on hepatic I/R-induced tissue damage by measurement of biochemical parameters in liver homogenates (MDA levels and CAT activities) and serum (ALT, AST and LDH activities).

## Materials and methods

### Animals

Twenty-eight adult male Sprague-Dawley rats (weighing 240–290 g) were provided from TICAM (Medical and Surgical Experimental Research Centre, Eskisehir Osmangazi University) and housed in polycarbonate cages in an air-conditioned room (22 ± 2 °C) with a 12 h light:12 h dark cycle (0700 on, 1900 hours off); standard rat feed and water were provided ad libitum. The rats were allowed to acclimatize to the laboratory environment for 7 days before the start of the experiment.

All procedures were conducted in conformity with the Institutional Ethical Committee for Animal Care and use at Eskisehir Osmangazi University (protocol number: 06-28/28) and the international guidelines on the ethical use of animals (NIH publications no: 80-23).

### Drug and reagent

Lycopene was purchased from Sigma (St. Louis, MO, USA; catalogue number: L9879-1 MG).

### Surgery

Under xylazine (10 mg/kg bw) and ketamine (70 mg/kg bw) anaesthesia, a minimal laparotomy was made using minimal dissection. Total hepatic ischemia was induced for 45 min by clamping the hepatic artery, the portal vein, and the bile duct using a vascular clamp. During the period of ischemia 0.5 ml of saline was given intraperitonally and then subjected to reperfusion for 60 min (Sener et al. 2003).

### Experimental groups

A total of 28 rats were divided into 4 groups (n = 7 per group); Group I: Normal control animals (NC), Group II: I/R control animals (IR-C), Group III: I/R animals given lycopene at the dose of 2.5 mg/kg bw (LYC-2.5) Group IV: I/R animals given lycopene at the dose of 5 mg/kg bw (LYC-5). Lycopene dissolved in olive oil (2 ml/kg volume) was injected intraperitonally 60 min before ischemia. Group II (I/R) was injected with olive oil (2 ml/kg volume) by the same way.

### Biochemical analysis

In this study, biochemical investigations were made in liver tissue and serum. The rats were sacrificed at 60 min of reperfusion by slight ether anaesthesia. Trunk blood samples were collected by cardiac puncture. As markers of hepatocellular injury serum ALT, AST and LDH levels were determined to assess liver functions. ALT, AST and LDH levels in serum were immediately measured with a commercial kit (Biolabo, Maizy, France) using an auto analyzer (Crony Instruments, Airone 200 RA, Rome, Italy). The serum ALT, AST and LDH were expressed as “U/l”.

MDA was measured by thiobarbituric acid reaction as a lipid peroxidation product according to the method of Uchiyama and Mihara (1978). CAT activity was determined using ammonium molybdate-hydrogen peroxide reaction as described previously by Goth (1991). The amount of total protein in tissue was measured with a total protein kit that was prepared according to the Biuret method. MDA, CAT, and total protein absorbance were determined by means of Shimadzu UV-1601 digital spectrophotometer (Shimadzu Corp. Kyoto, Japan). The hepatic MDA level and CAT activity were expressed as “nmol/g protein” and “KU/g”, respectively.

### Statistical analysis

Results were expressed as mean ± SD (Standard Deviation). The intergroup variation was measured by one-way analysis of variance (Anova) followed by Tukey’s test to assess the significance. Statistical significance was considered at “*p* < 0.05”. The statistical analysis was done using the SPSS Statistical Software version 12.

## Results

As shown in Table 1; the use of LYC at the doses of 2.5 and 5 mg/kg bw significantly reduced (*p* < 0.05) the ALT, AST, LDH and MDA values after hepatic ischemia/reperfusion (ALT 11.39 and 23.42 %, AST 8.62 and 28.21 %, LDH 19.50 and 33.08 %, MDA 15.75 and 26.14 %, respectively) when compared to IR-C group. In addition, these improvements materialized partially and dose-dependently.

**Table 1:** Effects of ischemia–reperfusion and its treatment with lycopene (at the doses of 2.5 and 5 mg/kg bw) in the serum ALT, AST, LDH and/or on hepatic MDA and CAT values

Groups^×^	ALT (U/L)	AST (U/L)	LDH (U/L)	MDA (nmol/g protein)	CAT (KU/g)
NC	47.32 ± 5.24	69.08 ± 5.83	125.42 ± 14.78	15.87 ± 1.02	6.79 ± 1.61
IR-C	386.40 ± 16.48^a^	416.47 ± 19.51^a^	858.38 ± 50.13^a^	30.03 ± 1.30^a^	3.58 ± 1.25^a^
LYC-2.5	342.36 ± 34.95^a, b^	380.54 ± 23.50^a, b^	690.97 ± 13.17^a, b^	25.30 ± 1.51^a, b^	5.80 ± 0.74^a, b^
LYC-5	295.89 ± 28.44^a, b, c^	298.97 ± 22.75^a, b, c^	574.40 ± 32.34^a, b, c^	22.18 ± 1.84^a, b, c^	6.82 ± 0.76^b, c^

^**×**^For details see “Materials and methods” section. Data are mean ± SD values (n = 7). *p* < 0.05, significantly different from ^a^ NC group, ^b^ IR-C group and ^c^ LYC-2.5 with LYC-5 groups by Tukey’s multiple range tests

The same benefits with LYC at the doses of 2.5 and 5 mg/kg bw were observed by CAT activities. The CAT activities were significantly increased (*p* < 0.05) in the LYC treated groups at the doses of 2.5 and 5 mg/kg bw (62.01 and 90.50 %, respectively) as compared to the IR-C group. Interestingly, these improvements materialized totally and dose-independently (Table 1).

Treatment of LYC at the doses of 2.5 and 5 mg/kg bw markedly improved the ALT, AST, LDH, CAT and MDA values as compared to the IR-C group values (*p* < 0.05). Especially; with respect to the CAT levels, the values of the LYC tretament groups were closer to those of the NC group (Table 1).

## Discussion

Considerable evidence shows that ROS are involved in the pathogenesis of hepatic I/R injury (Song et al. 2009). ROS changes carbohydrates, lipids and proteins, as well as inactivates enzymes and transporters, damages DNA and the transcriptional machinery, and initiates the chain of reactions that peroxidize polyunsaturated fatty acids in membrane phospholipids (Somi et al. 2009). Peroxidation of membrane lipids can affect membrane fluidity and cell compartmentation, which can further lead to cell lysis. In fact, lipid peroxidation is implicated in the pathogenesis of different liver injuries and subsequent liver fibrogenesis in humans and experimental animals. MDA is a major reactive aldehyde that appears during the peroxidation of the polyunsaturated fatty acids of biological membrane. Therefore, the hepatic content of it can be used as an indicator of liver tissue damage (Bi et al. 2008). Interestingly, in our study, LYC treatment caused a marked inhibition in MDA production (Table 1). This decrease indicated that lipid peroxidation of liver tissue and cellular injury was reduced. The protective effect of LYC is most likely due to its ability to scavenge the free radicals thereby limiting liver damage.

For protection against the ROS generated during I/R injury, liver tissues have developed an antioxidant defense system. (Yu et al. 2005). Enzymes such as CAT and SOD, act to remove oxygen free radicals (Polat et al. 2008). CAT subsequently converts hydrogen peroxide to water and oxygen. Removal of H_2_O_2_ by CAT is significant in preventing lipid peroxidation of membranes by OH radical. The role of it may be more important during hyperoxic conditions than in normoxic conditions (Yu et al. 2005). In our study, we found that LYC at the doses of 2.5 and 5 mg/kg bw administrated 60 min before initiation of ischemia caused a substantial increasing in the I/R induced decrease in CAT. Treatment of LYC was able to protect against the decrease in the activity of this enzyme in the I/R group (Table 1). These results demonstrate that LYC can be used as a drug against hepatic damage induced by I/R in favour of CAT activity.

ALT, AST, and LDH are generally used as markers of cellular damage following hepatic I/R injury. In some studies, it has been shown that the serum concentrations of these enzymes increase in proportion with the duration of ischemia (Kucuk et al. 2009). In our study, treatment of LYC at the doses of 2.5 and 5 mg/kg bw decreased markedly the serum ALT, AST and LDH levels as compared to IR-C group levels (*p* < 0.05) (Table 1). This result suggests that LYC decreases hepatic cellular damage in favour of ALT, AST and LDH activities.

In conclusion, the results of our study demonstrate that lycopene has a protective effect against hepatic I/R damage. The severity of hepatic I/R damage, as evidenced by increased levels of AST, ALT, LDH, MDA and decreased level of CAT, was alleviated by LYC treatment. LYC with its potent free radical scavenging and antioxidant properties seems to be a promising agent in protecting liver against ischemia/reperfusion injury.
